# Carotid artery dissection recanalization: imaging modalities, influencing factors, and therapeutic perspectives

**DOI:** 10.3389/fneur.2025.1624698

**Published:** 2025-07-08

**Authors:** Tao Li, Wenjing Lan, Xuanxiao Zhang, Shuo Yin, Pengfei Sun, Xin Chen, Hongwei Zhou

**Affiliations:** Department of Radiology, The First Hospital of Jilin University, Changchun, China

**Keywords:** carotid artery dissection, revascularization, diagnostic imaging, treatment outcome, decision making

## Abstract

Carotid artery dissection (CAD) is a rare cause of ischemic stroke, and its prognosis is often poor. If not diagnosed and treated in time, it may lead to serious complications such as intracranial stroke and even death. Accurate diagnosis of CAD, formulation of reasonable treatment plans, and prediction of vascular recanalization are crucial for improving the prognosis of patients. However, there is currently a lack of large-scale randomized controlled trials to provide guidance for clinical practice, and the industry has not yet reached a unified consensus on the standardized diagnosis and treatment of CAD. Therefore, this article reviews the imaging examination methods for recanalization of CAD, the analysis of related factors affecting recanalization, and the methods of recanalization treatment, and combines the latest research progress to provide a perspective on the recanalization of carotid artery dissection, aiming to provide a reference basis for the precise diagnosis and treatment of CAD recanalization.

## 1 Introduction

Carotid artery dissection (CAD) is a clinically significant cerebrovascular disease characterized by the tearing of the intima or rupture of the carotid artery wall (including the internal carotid artery and vertebral artery), leading to the formation of intramural hematoma. This pathological process can further cause severe complications such as intraluminal thrombosis, vascular stenosis, occlusion, or pseudoaneurysm (1). Notably, CAD accounts for only about 2% of all ischemic strokes (2), but its proportion significantly increases to 15%-25% in young and middle-aged stroke patients under 50 years old (3–5). Epidemiological studies show that the overall incidence of CAD is approximately 4.69 per 100,000 person-years, with the incidence of internal carotid artery dissection and vertebral artery dissection being 2.43 and 2.01 per 100,000 person-years, respectively (6). This disease is characterized by high mortality, high disability rate, and high recurrence rate, making it a significant public health issue threatening the health of the nation.

Current research indicates that the majority of CAD cases are spontaneous, but it is worth noting that approximately 90% of traumatic dissections are caused by minor trauma, including neck massage, weightlifting, yoga, childbirth, and other daily activities (7). Additionally, multiple studies have identified various potential risk factors, such as recent infection, pregnancy status, oral contraceptive use, smoking history, migraine, elongated styloid process, vascular anatomical variations, and genetic susceptibility (5, 8–10). Interestingly, recent studies have even found an association between higher education levels and CAD-related ischemic stroke in young people (11). However, the relationship between these factors and vascular recanalization after CAD remains to be further confirmed. In clinical practice, due to the relative rarity of CAD, the existing evidence mainly comes from case reports and case series studies, lacking the support of large-scale randomized controlled trials, which has led to the absence of a unified consensus on diagnosis and treatment at the international level (7). With the rapid development of modern imaging techniques, the detection rate of CAD has significantly increased, which poses higher requirements for the diagnostic and therapeutic decision-making abilities of clinicians (12). Currently, the commonly used treatment strategies for CAD include intraluminal thrombolysis, antiplatelet/anticoagulant drug therapy, and endovascular interventional therapy. However, clinical observations have found that some patients have an unsatisfactory response to the existing treatment regimens (1). As a key indicator for evaluating treatment efficacy, the recanalization rate is influenced by multiple factors, but systematic research on the influencing factors of recanalization after CAD is still limited.

**Figure 1:**
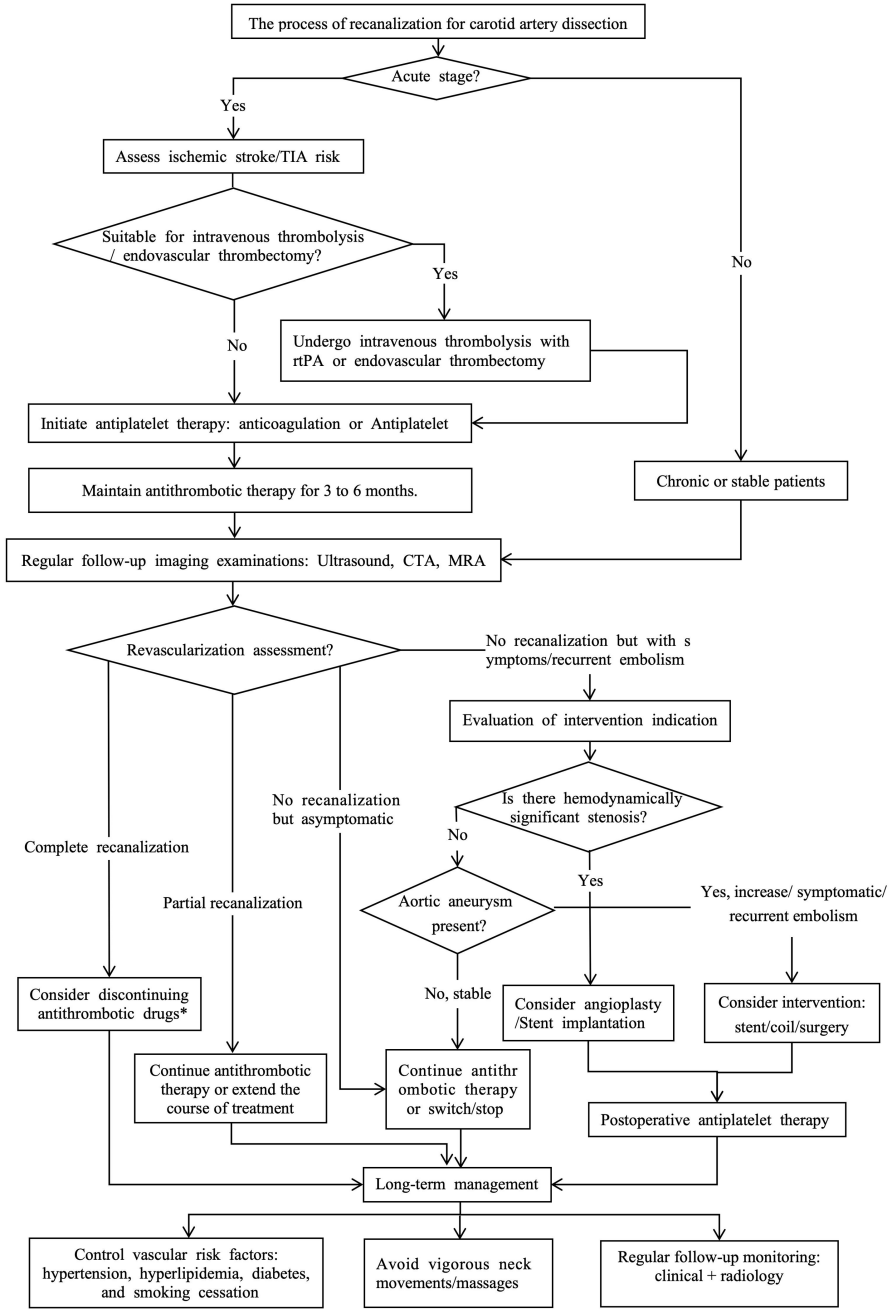
The process of recanalization for carotid artery dissection.

Based on the current research status, this article aims to comprehensively analyze the existing evidence on the imaging examination methods, influencing factors, and treatment decisions for CAD recanalization, and combine the latest research results to provide a perspective on CAD recanalization, in order to optimize clinical practice and guide future research (Figure 1).

## 2 Imaging examination for cervical artery dissection (CAD) revascularization

Cervical artery dissection has a relatively low incidence in the general population, but its actual prevalence may be higher due to asymptomatic or mildly symptomatic patients who do not seek medical attention. Between 2002 and 2020, the incidence of CAD increased nearly fourfold over 19 years, which may reflect advancements in imaging technology (6, 13), enabling more precise diagnosis of CAD patients through imaging modalities. This section describes four mainstream imaging diagnostic methods for CAD and their respective advantages, limitations, and clinical considerations.

### 2.1 CT angiography (CTA)

**Figure 2:**
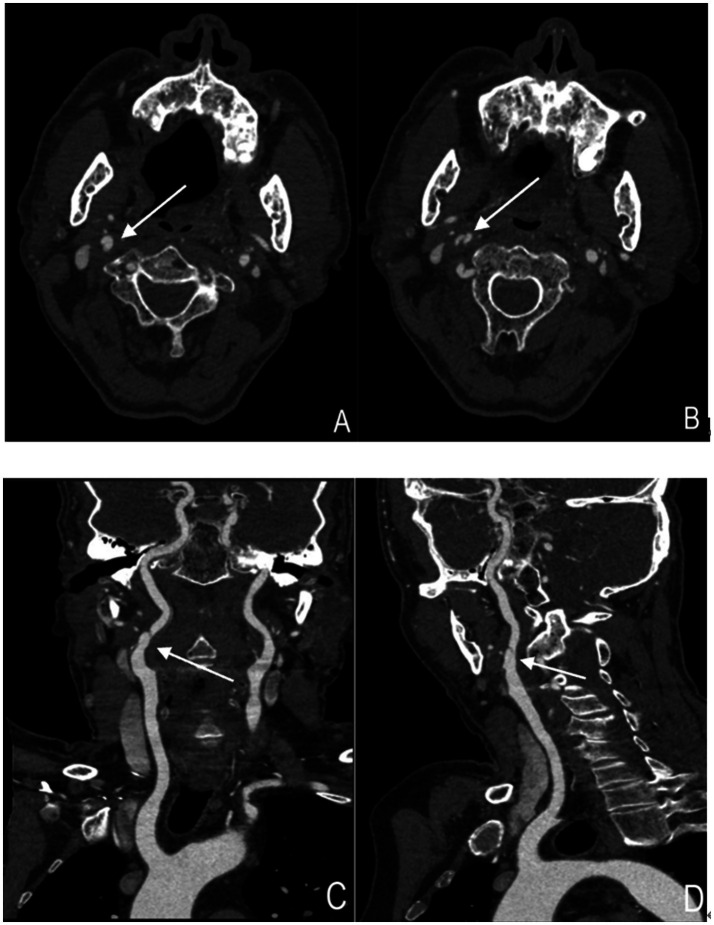
Cervical artery dissection showing arterial dissection-related vascular changes (right internal carotid artery). **(A,B)** Axial images of the right internal carotid artery. The structure indicated by the arrow represents the "double lumen sign," where the intimal flap divides the vascular lumen into two parts. **(C,D)** Coronal and sagittal images of the right internal carotid artery, respectively. The structure indicated by the arrow represents the "double lumen sign".

Cervical artery dissection has become one of the preferred imaging modalities for CAD diagnosis due to its rapid acquisition speed, high spatial resolution, and wide applicability (14). CTA demonstrates superiority over MRA in visualizing intimal flaps, dissecting aneurysms, and vascular lumen stenosis (15)(Figure 2).Particularly, the application of photon-counting CTA enables more precise depiction of dissection flaps, false lumens, and pseudoaneurysms (16). Given the smaller diameter of vertebral arteries and their proximity to cervical bony structures, CTA exhibits enhanced diagnostic performance for vertebral artery dissection (VAD) (17, 18), with sensitivity and specificity comparable to Digital subtraction angiography (DSA) (19). Furthermore, CT perfusion imaging (CTP) provides hemodynamic information about distal intracranial circulation in acute dissection cases, aiding patient selection for mechanical endovascular reperfusion therapy, leading to increasingly combined use of CTP with CTA in auxiliary diagnosis (12). The primary disadvantages of CTA involve radiation exposure and contrast agent administration, necessitating cautious use in patients with contrast allergies, renal insufficiency, as well as children and pregnant women (20). Additionally, inaccurate contrast injection timing and the presence of metallic implants can compromise image quality and diagnostic accuracy.

### 2.2 Magnetic resonance angiography (MRA)

Magnetic resonance angiography represents a non-invasive multi-parametric imaging technique with high soft tissue and spatial resolution, lacking radiation exposure, and serving as a crucial modality for CAD evaluation. MRA, in combination with axial fat-suppressed T1-weighted imaging, better identifies small intramural hematomas (21). However, MRA exhibits lower sensitivity in the early stages of CAD (22, 23). Diffusion-weighted imaging (DWI) application compensates for this limitation, with studies demonstrating DWI's capability to rapidly detect abnormalities during the initial phase of CAD and effectively assess intramural hematoma length (24, 25). Compared to DSA, MRA achieves a diagnostic sensitivity of 95% for cervical artery dissection, although its sensitivity for vertebral artery dissection remains relatively lower (26, 27). Additional studies have reported that susceptibility-weighted imaging (SWI) represents a highly sensitive imaging sequence with advantages in diagnosing vertebral artery dissection (28, 29). Nevertheless, MRI possesses certain limitations and contraindications, including absolute contraindications for patients with cardiac pacemakers, metallic implants, and claustrophobia, along with high costs, prolonged examination times, and susceptibility to artifacts, restricting its widespread clinical application.

### 2.3 High-resolution magnetic resonance imaging (HR-MRI)

**Figure 3:**
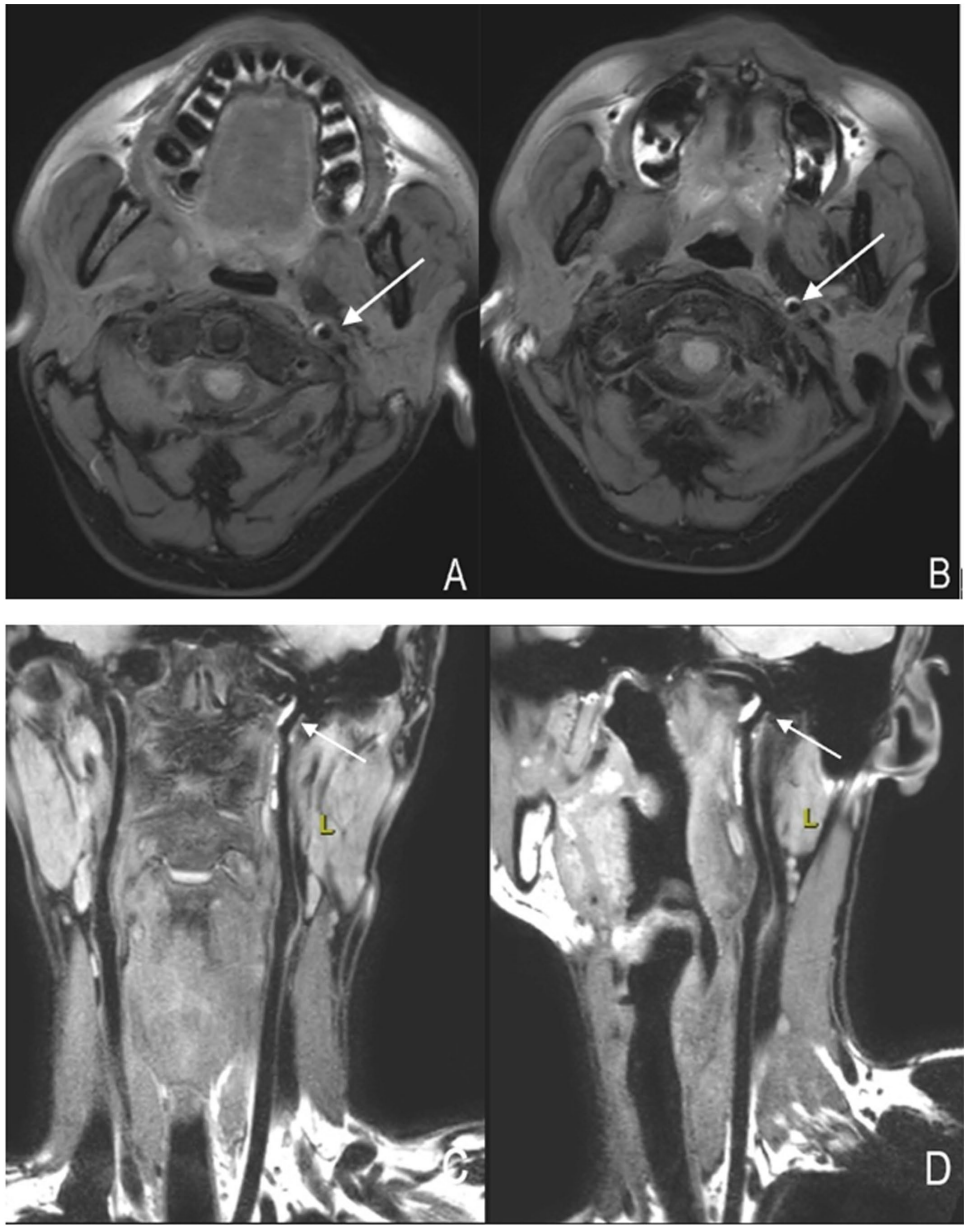
High-resolution magnetic resonance imaging showing arterial dissection-related vascular changes. **(A,B)** Axial images of the left internal carotid artery. The crescent-shaped high signal indicated by the arrow represents an "intramural hematoma." **(C,D)** Coronal and sagittal images of the left internal carotid artery, respectively. The high-signal structure indicated by the arrow represents an "intramural hematoma".

High-resolution magnetic resonance imaging vessel wall imaging technology, based on black-blood imaging sequences, employs presaturation pulses to suppress intraluminal blood flow signals, enabling clear visualization of cervical artery wall and lumen structures, significantly improving the detection rate of intramural hematomas (22, 30–33) (Figure 3). Research indicates that high-resolution MRI vessel wall imaging not only clearly visualizes collapsed vascular walls with occlusive thrombi and occlusion lengths in cases of cervical artery occlusion but also demonstrates better consistency with DSA in detecting tandem lesions and chronic occlusions of the internal carotid artery (ICA) (34, 35). Moreover, high-resolution vessel wall imaging holds significant value in early risk assessment and prognostic follow-up of CAD patients. Wu et al. (36) found that irregular surfaces and intraluminal thrombi on high-resolution imaging correlate with stroke occurrence in patients with cervical-carotid artery dissection (CCAD). Lee et al. (37) demonstrated that HR-MRI enables tracking of hematoma absorption processes and predicts dissection vessel recanalization based on changes in intramural hematoma signals. Hashimoto et al. (33) further emphasized that the temporal sequence signal characteristics of T1-weighted vessel wall imaging for intramural hematomas may serve as diagnostic imaging biomarkers for spontaneous healing within 3 months post-VAD onset. Additional studies report that HR-MRI vessel wall imaging outperforms DSA in diagnosing vertebral artery dissection (38). Given HR-MRI's superior visualization of vessel walls and demonstrated prognostic follow-up advantages, it is currently considered the most promising imaging modality for CAD diagnosis (39).

### 2.4 Ultrasound (US)

**Figure 4:**
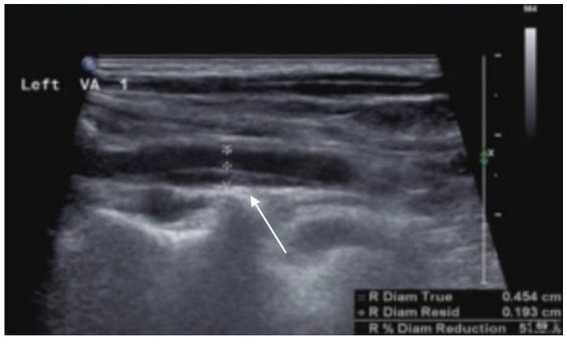
Arterial dissection is observed in the left vertebral artery. The posterior wall of the left vertebral artery shows an intramural hematoma, resulting in luminal narrowing, with a residual diameter of 1.9 mm and an original diameter of 4.5 mm.

Head and neck vascular US represents a safe, economical, and non-invasive examination method, although its accuracy significantly depends on operator experience and correlates with lesion severity and dissection location. Carotid ultrasonography can observe the lumen and wall structures, which can reveal signs such as the "dual lumen sign," "intramural hematoma," and "intimal flap"(Figure 4).US frequently serves in follow-up evaluations to identify vascular recanalization and remodeling. During the early stages of CAD (particularly within the first 4 weeks), US proves crucial for assessing clinical status and monitoring vascular recanalization (40). Doppler US effectively evaluates in-stent restenosis post-carotid stenting, with pre-discharge US examinations confirming stent patency (41). Research suggests that US monitoring significantly aids in identifying retrograde thrombosis formation and recanalization status in internal carotid artery dissections (42). Reported diagnostic sensitivities for vertebral artery dissection reach 92%, while for cervical artery dissections causing only localized symptoms, sensitivity decreases to 69% (43), indicating potential missed diagnoses of mild stenotic dissections. However, for severe stenotic dissections inducing hemodynamic changes, US achieves sensitivities as high as 96%. Therefore, for clinically concerning cases with negative US results but persistent clinical suspicion, further MRA or CTA examinations are recommended.

### 2.5 Digital subtraction angiography (DSA)

**Figure 5:**
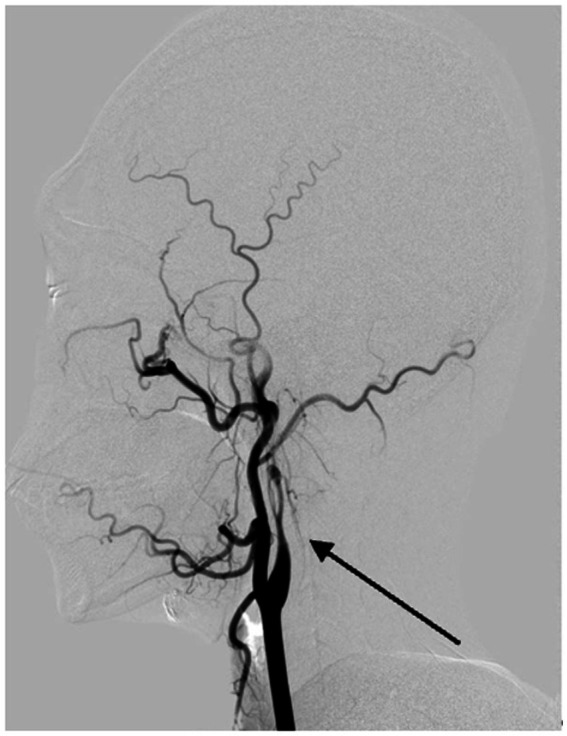
Digital subtraction angiography reveals vascular changes due to arterial dissection in the left internal carotid artery. The DSA image clearly demonstrates evidence of dissection in the left internal carotid artery, with the arrow pointing to a structure consistent with the 'flame sign,' characteristic of this condition.

Long regarded as the gold standard for CAD diagnosis, DSA offers high spatial and temporal resolution, enabling direct visualization of luminal structures and dynamic observation of pathological vascular blood flow patterns, as well as evaluating collateral circulation and hemodynamic compensation (Figure 5). However, as an invasive procedure, DSA entails high costs, prolonged durations, and lacks assessment of vascular wall structures. In cases of subadventitial dissections without significant luminal narrowing, DSA may yield false negatives (44). Current guidelines recommend avoiding DSA as a first-line diagnostic tool, reserving it for patients with discordant MRA and CTA findings (1).

## 3 Analysis of factors influencing cervical artery dissection revascularization

Vascular recanalization closely correlates with the prognosis of CAD patients. As a key evaluation metric in CAD treatment reflecting therapeutic efficacy, improving vascular recanalization rates remains a clinical focus. This section explores and analyzes factors associated with vascular recanalization imaging, providing a basis for personalized patient management.

### 3.1 Hypertension

Hypertension has long been recognized as an independent risk factor for CAD (45), although its impact on CAD vascular recanalization remains controversial. A large-scale multicenter cohort study on patients with Cervical Artery Dissection and Ischemic Stroke Patients (CADISP) conducted in 2011 found that the prevalence of hypertension was higher among patients with CAD (46). Numerous studies (47–49) indicate that hypertension correlates with lower vascular recanalization rates, hypothesizing that elevated blood pressure accelerates endothelial injury, reduces arterial wall elasticity and permeability, promotes atherosclerosis, increases thrombus burden, and hinders complete vascular recanalization. Wadhwa et al. (49) noted that while hypertension constitutes a CAD risk factor, it is not the sole determinant influencing recanalization, with results potentially confounded by age-related factors as hypertensive patients tend to be older. Conversely, other studies (50–53) yielded contradictory results, showing no significant correlation between hypertension and vascular recanalization rates, possibly due to younger patient inclusion compared to studies reporting such associations. Objective validation through further research remains necessary to clarify hypertension's role in CAD recanalization.

### 3.2 Time

Revascularization in cervical artery dissection typically occurs during the early stages post-symptom onset, with an average time to complete or near-complete recanalization approximately 4.7 months (54). CAD represents a highly dynamic process, exhibiting observable imaging changes within short timeframes, including stenosis regression and occlusion recanalization in most cases (22, 55). Research defines the acute phase as within 14 days post-arterial dissection onset (22, 56), with the highest risk of recurrent transient ischemic attacks or strokes within 14 days (54, 57). Huang et al. (51) identified time from onset to presentation ≤14 days as a critical factor for complete vascular recanalization, with most CAD patients achieving favorable outcomes post-treatment. Liu et al. (34) found that initial ischemic events within 3 months serve as independent predictors of carotid artery occlusion recanalization, corroborating the correlation between onset timing and vascular recanalization, although further large-scale cohort randomized trials are required for confirmation.

### 3.3 Intramural hematoma

Intramural hematomas frequently occur in CAD patients' vessels. Huang et al. (51) demonstrated a positive correlation between intramural hematomas and complete vascular recanalization, with higher complete recanalization rates observed in hematoma-type CAD patients. Some studies suggest that stabilized intramural hematomas gradually transforming into fibrotic tissue during the acute phase result in lower vascular remodeling potential and reduced recanalization likelihood (58). Vicenzini et al. (42) posited that retrograde thrombosis in the internal carotid artery may relate to persistent occlusions and partial recanalization, with intracarotid thrombus remodeling potentially extending over 2 years. Additional research indicates that changes in intramural hematomas reflect early dynamic alterations in dissections, aiding in predicting vascular recanalization outcomes (33, 59).

### 3.4 Vascular occlusion

Vascular occlusions caused by cervical artery dissections significantly correlate with increased stroke risks, adverse functional outcomes, and irreversible vascular changes (60–62), rendering the relationship between vascular occlusion and recanalization rates highly pertinent. A 2008 prospective multicenter study investigating predictors of Symptomatic Intracranial Atherosclerotic Disease (sICAD) recanalization found that complete occlusions reduced the likelihood of complete recanalization (53). Huang et al. (51) reached similar conclusions, identifying vascular occlusion as a risk factor for incomplete recanalization, with lower complete recanalization rates observed in occluded patients. Zhou et al. (63) found that true lumen stenosis <90% correlated with complete recanalization. Other studies reported lower recanalization rates in occluded compared to stenotic vessels (3, 55), with partial occlusions exhibiting approximately double the recanalization rate of complete occlusions (64, 65). Some scholars observed that occluded or near-occluded vessels at presentation rarely recanalize (54), while Arauz et al. (50) found no differences in complete recanalization rates among completely occluded vessels, suggesting substantial challenges in recanalizing fully occluded vessels. Overall, vascular occlusion significantly correlates with reduced recanalization rates.

### 3.5 Treatment modalities

Antithrombotic therapy represents the primary treatment for CAD, although debates persist regarding the comparative effectiveness of anticoagulation versus antiplatelet therapy in CAD vascular recanalization. Some scholars argue that anticoagulation may expand intramural hematomas, hindering vascular recanalization (58). Conversely, Nedeltchev et al. (53) observed a trend toward higher complete recanalization rates in anticoagulated patients, albeit statistically insignificant. A multicenter prospective randomized focus trial Carotid Artery Dissection in Stroke study (CADISS) found no significant difference in recanalization rates at 1 year in patients with coronary artery disease treated with anticoagulation and antiplatelet therapy (66), consistent with Huang et al.'s findings (51). These results suggest that both antithrombotic and anticoagulant treatments serve as viable options with low complication risks in CAD patients. Considering the increased bleeding risks associated with anticoagulation, antithrombotic drug selection should follow individualized treatment protocols tailored to patient-specific conditions.

### 3.6 Local symptoms and signs

Nedeltchev et al. (53) found that CAD patients presenting with local symptoms and signs (including head and neck pain, Horner syndrome, cranial nerve palsies) exhibited significant correlations with vascular recanalization rates. Huang et al. (51) noted a trend toward higher complete recanalization rates in CAD patients presenting solely with local symptoms, although statistically insignificant. Additional research (46, 52) identified obesity as a factor contributing to poor CAD outcomes, potentially due to concomitant hypertension and hyperlipidemia exacerbating vascular endothelial injury in obese patients.

### 3.7 Genetic factors

The first genome-wide association study on CAD revealed that the Phosphatase and Actin Regulator 1(PHACTR1) gene's rs9349379-A allele associated with increased risks of coronary artery dissection and hypertension (67). Le Grand et al. (68) employed Mendelian randomization analysis to explore causal relationships between vascular risk factors (including blood pressure, lipids, diabetes) and CAD risks/recurrences, finding that genetically predicted higher systolic and diastolic pressures correlated with increased CAD risks, while genetic proxies for antihypertensive beta-blockers reduced CAD risks (69). These findings underscore the importance of monitoring blood pressure in all CAD patients and recommend beta-blocker therapies (69), suggesting that genetic factors may represent a potentially crucial determinant influencing CAD recanalization.

## 4 Decision-making in cervical artery dissection revascularization therapy

Current treatment options for cervical artery dissection encompass intravenous thrombolysis, antiplatelet and anticoagulant therapies, endovascular interventional treatments, or surgical interventions, although clear boundaries between these therapeutic approaches remain undefined.

### 4.1 Intravenous thrombolysis

As an effective method for treating acute ischemic strokes, intravenous thrombolysis historically aimed to recanalize occluded vessels. However, thrombolytic therapy increases the risk of intracerebral hemorrhage. Although some studies have confirmed the safety of intravenous thrombolysis in CAD-induced acute ischemic stroke, the evaluation of its efficacy is still controversial. Two meta-analyses found that CAD-related stroke patients receiving intravenous thrombolysis exhibited safety profiles comparable to ischemic strokes from other causes, without increased risks of symptomatic intracranial hemorrhage (70, 71). Numerous observational studies reached similar conclusions (72–74). Regarding whether intravenous thrombolysis improves arterial outcomes in CAD patients, current research findings remain inconsistent. Engelter et al. (72) found no significant advantages of intravenous thrombolysis over non-thrombolytic treatments in CAD-related stroke patients. However, secondary analyses of the STOP-CAD study data revealed significant associations between IVT use and functional independence at 90 days in CeAD-induced AIS patients, suggesting improved functional outcomes with IVT (75). Previous studies also indicated similar efficacies of intravenous thrombolysis in CAD-related acute ischemic strokes compared to strokes from other causes. Future large-scale randomized controlled trials are necessary to guide clinical decision-making.

### 4.2 Anticoagulation and antiplatelet therapies

Approximately 85% of ischemic strokes in CAD result from arterial-arterial embolism (76), prompting routine antithrombotic or anticoagulant therapies to mitigate thromboembolic risks. Substantial data support the safety and efficacy of anticoagulant and antiplatelet treatments in CAD patients, although consensus on therapeutic selection and duration remains elusive. Two multicenter randomized controlled trials, including CADISS and TREAT-CAD studies, compared the efficacies of antiplatelet and anticoagulant therapies. The CADISS study revealed no significant differences in stroke risk prevention within 3 months between antiplatelet and anticoagulant treatments in CAD patients, despite higher stroke frequencies in the antiplatelet group and one major bleeding event in the anticoagulant group (77). One-year follow-ups showed no differences in recurrence or recanalization rates among CAD patients (66). The TREAT-CAD study failed to demonstrate the non-inferiority of aspirin over vitamin K antagonists, with all ischemic strokes occurring in the aspirin group and the sole major bleeding event in the VKA group, highlighting the importance of early antithrombotic therapy initiation in CAD management (78). These studies suggest that both antithrombotic and anticoagulant treatments represent viable options in CAD patients with low complication risks. Janquli et al. (79) reached similar conclusions, demonstrating favorable clinical and anatomical outcomes with both treatments, without significant differences between them. Furthermore, increasing evidence suggests that combinations of aspirin or clopidogrel may more effectively prevent early recurrent stroke risks (66). Wadhwa et al. (49) found potential benefits of triple therapy (dual antiplatelet therapy and heparin) in promoting vascular recanalization during the early stages of dissection.

The optimal duration of antithrombotic therapy remains controversial and requires further exploration through more randomized controlled studies. A multicenter, observational, retrospective international study (STOP-CAD study) found that anticoagulation therapy did not have a significant advantage in reducing the risk of ischemic stroke, but it might be beneficial in patients with occlusive dissection. 87% of ischemic stroke events occurred within 1 month of dissection diagnosis. If anticoagulation therapy is chosen, switching to antiplatelet therapy within 180 days is reasonable (80). Pezzini et al. (81) compared the risk of ischemic stroke in patients who stopped and continued antithrombotic therapy. The results suggested that stopping antithrombotic therapy after 6 months did not increase the risk of cerebral ischemia during the follow-up period. Wadhwa et al. (49) demonstrated that long-term use of antiplatelet drugs (>6 months) or anticoagulation therapy did not affect recanalization status.

Current guidelines recommend anticoagulation for CAD patients with high-risk factors such as severe stenosis/occlusion or intraluminal thrombus formation and low bleeding risks, while favoring antiplatelet therapy for patients with higher bleeding risks. Antithrombotic therapies typically extend for 3-6 months, although whether to prolong treatment beyond 6 months should be individually determined.

### 4.3 Endovascular intervention or surgery

Endovascular interventional therapy or surgical treatment also represents crucial approaches for cervical artery dissection (CAD) recanalization. Multiple studies (82–85) have compared the safety and efficacy of endovascular treatments versus intravenous thrombolysis in CAD-related acute ischemic strokes, demonstrating that endovascular therapies (including mechanical thrombectomy, angioplasty, and/or stent placements) exhibit better outcome trends, higher recanalization rates, and do not increase risks of symptomatic intracranial hemorrhage or early mortality. However, a multinational prospective cohort study revealed that although endovascular treatments achieved higher complete recanalization rates, they did not demonstrate superior functional outcomes compared to intravenous thrombolysis (IVT) in CAD patients with acute ischemic stroke (AIS) and large vessel occlusion (LVO) (83).

Additional scholars (86–88) independently evaluated the safety and efficacy of endovascular thrombectomy, reaching similar conclusions that mechanical thrombectomy enhances favorable outcomes and success rates, improving prognoses. Compared to medical treatments and non-CAD stroke patients, endovascular thrombectomy exhibited no significant differences in symptomatic hemorrhage or mortality rates. However, considering the relatively higher rates of symptomatic hemorrhage and mortality, the safety of thrombectomy requires further verification. Scopelliti et al. (89) further emphasized that ensuring sustained internal carotid artery patency post-thrombectomy significantly correlates with better functional outcomes at 3 months.

Moreover, studies indicate that for CAD patients unresponsive to medical treatments, vascular stent placements and surgical interventions prove feasible and effective (90, 91). Nevertheless, a Cochrane systematic review found no randomized controlled trials (RCTs) or controlled clinical trials (CCTs) supporting additional benefits of surgical or endovascular treatments over antithrombotic therapies when the latter prove ineffective (92).

Endovascular treatments appear safe and effective in CAD-induced AIS patients. Future research should conduct relevant RCTs to further explore the safety and efficacy of endovascular therapies in CAD patients and determine optimal treatment strategies.

## 5 Issues and discussion

Cervical artery dissection represents a rare cause of stroke but constitutes a primary etiology among young and middle-aged stroke patients. Digital subtraction angiography (DSA) historically served as the imaging gold standard for CAD diagnosis due to its direct visualization of vascular luminal structures. However, considering DSA's invasiveness, it remains unsuitable for all patients (93). In contrast, ultrasound, computed tomography angiography (CTA), and magnetic resonance angiography (MRA) offer non-invasive alternatives. Head and neck vascular ultrasound examinations, characterized by low costs and simple operations, frequently serve in arterial remodeling follow-up studies but may lead to missed diagnoses (43), necessitating subsequent CTA or MRA examinations. Both CTA and MRA demonstrate superior advantages in visualizing luminal structures, achieving high sensitivity and specificity in CAD diagnosis (19, 27). However, the introduction of contrast agents and inherent equipment limitations somewhat restrict their clinical applications. Recent advancements in imaging technology have enabled high-resolution vessel wall magnetic resonance imaging (HR-VW-MRI) to non-invasively and directly display hematoma signal locations, sizes, and other characteristics within dissected vessel walls, significantly improving CAD detection rates. Particularly, artificial intelligence compressed sensing technology (CS-AI) enhances VW-MRI image quality and diagnostic efficiency, demonstrating promising applications in diagnosing atherosclerotic vascular diseases (94). Given that CS-AI-integrated HR-VW-MRI offers shortened scanning times, superior vascular wall visualization, and vascular lesion assessments, we reasonably anticipate achieving high-quality CAD vessel wall imaging within shorter durations in the near future. All kinds of imaging examinations have their advantages and limitations, and we need to choose the most appropriate imaging method based on their characteristics (Table 1).

**Table 1:** A comparative analysis of imaging examination methods for carotid artery dissection.

Modaity	Indications	Advantages	Limitations
CTA	Acute evaluation, emergency screening, bone assessment	1. Rapid acquisition (first-line in emergencies) 2. High spatial resolution (intimal flap, intramural hematoma) 3. 3D reconstruction 4. Widely available	1. Requires iodinated contrast (risk in renal impairment) 2. Radiation exposure 3. Lower sensitivity for slow flow/complex dissections
MRA	Non-acute phase, follow-up, contrast allergy	1. No radiation 2. No iodinated contrast needed (TOF technique) 3. Excellent soft-tissue contrast (intramural hematoma)	1. Longer scan time (less ideal for acute cases) 2. Flow-related artifacts 3. Insensitive to calcifications
HR-MRA	Small dissections, detailed intimal flap assessment, research	1. Ultra-high resolution (0.1–0.3 mm) 2. Superior visualization of subtle lesions 3. No radiation	1. Limited to advanced MRI systems 2. Longer acquisition time 3. Low clinical availability
DSA	Gold standard, pre-interventional confirmation, endovascular therapy	1. Best temporal resolution (dynamic flow assessment) 2. Enables simultaneous treatment (e.g., stenting) 3. Highest diagnostic accuracy	1. Invasive (puncture risks, 0.5–1% stroke risk) 2. High cost 3. Requires expert operators
US	Initial screening in suspected CAD; Follow-up monitoring of recanalization; Low-risk or stable patients	1. Non-invasive, no radiation/contrast 2. Bedside availability (quick assessment) 3. Real-time hemodynamic evaluation (flow patterns, stenosis) 4. Cost-effective	1. Operator-dependent (variable accuracy) 2. Limited visualization of distal ICA/vertebral arteries 3. Lower sensitivity for small dissections/intramural hematoma 4. Cannot assess intracranial extension

Thromboembolism represents the most common cause of stroke in CAD patients (76), with CAD frequently manifesting as thromboembolism or luminal stenosis/occlusion-induced transient ischemic attacks or acute ischemic strokes (7). achieving vascular recanalization is a pivotal determinant of CAD prognosis and serves as a key endpoint for evaluating therapeutic efficacy. Understanding the complex interplay of factors influencing CAD recanalization and translating this knowledge into tailored therapeutic strategies is therefore paramount for optimizing patient outcomes. Previous studies have identified several key factors potentially influencing CAD vascular recanalization, including hypertension, time from symptom onset to presentation, the presence of an intramural hematoma (IMH), complete vascular occlusion, and the specific treatment modality employed. however, the precise pathways require further elucidation. Critically, synthesizing the literature reveals that these factors do not act in isolation but may interact, significantly shaping recanalization likelihood and informing treatment choices. Hypertension stands out as the predominant vascular risk factor specifically linked to CAD (95, 96). Proposed mechanisms by which hypertension may impact CAD recanalization include non-atherosclerotic arterial wall injury (97) and alterations in arterial wall elasticity and permeability (47).

Although it is known that hypertension treatment can significantly reduce the risk of first and recurrent strokes, there is no convincing evidence to support that one class of antihypertensive drugs is superior to another as a single therapy for secondary prevention in stroke patients. Although some antihypertensive drugs have neuroprotective effects, clinical data on the pre-treatment impact of antihypertensive drugs on the treatment outcomes of stroke patients remain controversial (98). Generally, untreated hypertension in patients with acute ischemic stroke is associated with poor treatment outcomes, and current guidelines recommend controlling hypertension during the acute phase of stroke (99). However, a multivariate analysis from a large multicenter study suggested that early antihypertensive treatment failed to reduce the probability of dependency or death at 90 days in ischemic stroke patients with a history of hypertension, but worsened the functional outcomes of patients without hypertension (100). These findings indicate that initiating antihypertensive treatment within the first week after an ischemic stroke does not bring significant benefits and may even increase the risk of functional dependency in patients without a history of hypertension. Hypertension is closely related to vascular recanalization, but the timing of treatment is crucial. The interaction between hypertension treatment and vascular recanalization deserves further study. The acute phase of aortic dissection lasts for 14 days. A retrospective study found that the recanalization rate of patients with a visit time less than 14 days was significantly higher than that of patients with a visit time greater than 14 days. Moreover, multivariate regression analysis revealed that a shorter time from symptom onset to visit (≤14 days) and coronary artery disease presenting as intramural hematoma (IMH type) were consistently positively correlated with recanalization (51, 56). This synergistic effect may reflect the dynamic evolution characteristics of the dissection flap/hematoma during the acute phase, during which early intervention may take advantage of its greater inherent instability or plasticity, thereby facilitating healing and recanalization.This finding strongly supports the clinical imperative for rapid diagnosis and initiation of therapy in suspected CAD.Complete vascular occlusion is robustly identified as an independent negative predictor of recanalization in multiple studies employing multivariable regression (101–103). This factor significantly outweighs others in predicting recanalization failure. The presence of occlusion should therefore trigger consideration of more aggressive therapeutic strategies (e.g., potential endovascular intervention, especially in specific scenarios) or heightened monitoring for complications and collateral assessment. Current evidence highlights that hypertension management, early diagnosis, IMH-type dissection recognition, and occlusion status assessment are key factors in CAD recanalization strategies, but further prospective studies with multivariable modeling are needed to strengthen evidence-based guidance.

While antithrombotic therapy is the primary initial CAD treatment and endovascular interventions (EVT) demonstrate efficacy in achieving recanalization and improving prognosis, particularly in symptomatic or perfusion-deficient patients (104–106), a balanced assessment necessitates careful consideration of treatment-specific risks and long-term outcomes. Intravenous thrombolysis (IVT) carries risks like symptomatic intracranial hemorrhage and potential dissection complications; conservative management faces bleeding risks and treatment failure; EVT introduces procedural risks (e.g., dissection extension, perforation) and, with stenting, long-term concerns of in-stent stenosis/thrombosis requiring intensive antiplatelet therapy (85, 107). Critically, long-term functional recovery—measured by outcomes like independence (mRS)—can be influenced by both treatment efficacy and associated complications. Therefore, developing individualized treatment plans based on patient-specific conditions (presentation, dissection characteristics, bleeding risk) and integrating knowledge of each modality's complication profile and functional outcome potential is paramount(Table 2). Implementing refined, classification-based diagnostic strategies to optimize this risk-benefit analysis is key to enhancing both recanalization success and long-term functional prognosis for CAD patients.

**Table 2:** A comparative analysis of treatment decisions for carotid artery dissection.

Methods	Indications	Timing	Risks	Efficacy
Antithrombotic Therapy (Anticoagulation/Antiplatelet)	First-line for most patients unless contraindicated (e.g., high bleeding risk).	Initiate immediately upon diagnosis; continue for 3-6 months.	Bleeding (GI, intracranial), lower risk with antiplatelets.	Prevents thromboembolism; ~70-90% effectiveness in reducing stroke risk.
Thrombolysis (IV-tPA)	Acute stroke with confirmed occlusion; use cautiously in CAD (case-by-case).	Acute ischemic stroke (<4.5 hours).	Dissection extension, hemorrhagic transformation, allergic reactions.	Rapid clot lysis but controversial in CAD (may worsen dissection).
Endovascular Therapy (Stenting/Thrombectomy)	Refractory cases, severe stenosis/occlusion, or recurrent ischemia on medication.	Acute large-vessel occlusion (<24 hours).	Vessel perforation, distal embolism, dissection progression, access-site hematoma.	High recanalization rates (>80%); effective for hemodynamically significant lesions.

## 6 Prospect

Currently, no universally recognized clinical classifications guide CAD treatments. Perry et al. (108) proposed the Borgess classification in 2013 based on imaging findings of intimal tears and blood flow impacts, categorizing dissections with intact intimal layers as Type I and those with intimal tears as Type II, observing that Type I predominantly presents ischemic symptoms while Type II exhibits more localized symptoms, with antithrombotic treatments post-Type I dissections showing higher healing probabilities than post-Type II. Zhou et al. (109) recently proposed a comprehensive classification system for cervical artery dissections (CAD), categorizing lesions into four distinct types based on angiographic features:

Type I: Intramural hematoma or dissection with <70% luminal stenosis.
Type II: Dissection with ≥70% luminal stenosis.
Type III: Dissecting aneurysm (vessel dilation exceeding 1.5× the normal diameter).
Type IV: Complete luminal occlusion, subdivided into:
Type IVA: Extracranial carotid occlusion.
Type IVB: Tandem occlusion (extracranial + intracranial involvement).

Their study further demonstrated that stable CAD patients benefit from antithrombotic therapy in reducing recurrent stroke risk. For Type II–IVA dissections, non-urgent endovascular treatment (EVT) may be considered as an alternative to antithrombotic therapy. Type IVB dissections often require urgent vascular intervention. Different classifications guiding surgical strategies have matured in aortic dissection progressions, suggesting potential benefits from more detailed CAD classifications and establishing distinct treatment methods based on classifications to achieve precise CAD management and enhance recanalization probabilities.

Advancements in artificial intelligence (AI) and radiomics technologies promise AI-driven predictions of CAD occurrence risks and recanalization scenarios, aiding clinical decisions. AI encompasses machine learning and deep learning (110), with deep learning as a significant branch of machine learning possessing robust feature extraction and generalization capabilities, extensively applied across various medical tasks (111–113). Current predictive models for carotid occlusion recanalization include Lin et al.'s (114) machine learning algorithm-developed pre-EVT and post-EVT models assessing recanalization risks, assisting clinicians in better evaluating patient prognoses. Radiomics, initially proposed by Dutch scholar Lambin (115), transforms digital medical images into mineable high-dimensional data (116). Radiomics captures tissue and lesion properties and their imaging changes during treatments; within sufficiently large datasets, it identifies unknown disease progression, progression, and treatment response biomarkers. The integration of radiomics and AI enables automated processing of larger datasets, emerging as a recent research focus. In carotid diseases, radiomics has been widely applied in plaque property assessments (117, 118). Image segmentations, modeling, and validations of CAD intramural hematomas, along with digital processing of arterial dissection vascular stenosis degrees, locations, and extents, could yield more meaningful clinical analyses, predicting post-dissection vascular recanalization probabilities. Furthermore, AI's enhanced ability to extract deep features from raw radiomics data advances explorations in carotid atherosclerosis, promoting early detection and diagnosis, risk stratification, predictive modeling, workflow efficiency improvements, and research advancements (119). Future radiomics and deep learning technologies may represent novel directions for CAD recanalization.

## 7 Conclusion

Cervical artery dissection results from interactions among risk factors, minor traumas, anatomical and congenital abnormalities, and genetic susceptibilities. Diagnosing CAD presents challenges both clinically and radiologically. Considering CAD prognoses and associated clinical and imaging prognostic factors, future research should conduct longitudinal and population-based observational studies, integrating advanced technologies to mitigate prognosis disparities arising from differing preferred treatment strategies.
